# Health at the borders: Bayesian multilevel analysis of women's malnutrition determinants in Ethiopia

**DOI:** 10.3402/gha.v9.30204

**Published:** 2016-07-04

**Authors:** Tefera Darge Delbiso, Jose Manuel Rodriguez-Llanes, Chiara Altare, Bruno Masquelier, Debarati Guha-Sapir

**Affiliations:** 1 Centre for Research on the Epidemiology of Disaster (CRED), Institute of Health and Society (IRSS), Université catholique de Louvain (UCL), Brussels, Belgium; 2 Action Contre la Faim, Paris, France; 3 Centre de Recherche en Démographie, Université catholique de Louvain (UCL), Louvain-la-Neuve, Belgium

**Keywords:** conflict, natural disaster, overweight, refugee, underweight

## Abstract

**Background:**

Women's malnutrition, particularly undernutrition, remains an important public health challenge in Ethiopia. Although various studies examined the levels and determinants of women's nutritional status, the influence of living close to an international border on women's nutrition has not been investigated. Yet, Ethiopian borders are regularly affected by conflict and refugee flows, which might ultimately impact health.

**Objective:**

To investigate the impact of living close to borders in the nutritional status of women in Ethiopia, while considering other important covariates.

**Design:**

Our analysis was based on the body mass index (BMI) of 6,334 adult women aged 20–49 years, obtained from the 2011 Ethiopian Demographic and Health Survey (EDHS). A Bayesian multilevel multinomial logistic regression analysis was used to capture the clustered structure of the data and the possible correlation that may exist within and between clusters.

**Results:**

After controlling for potential confounders, women living close to borders (i.e. ≤100 km) in Ethiopia were 59% more likely to be underweight (posterior odds ratio [OR]=1.59; 95% credible interval [CrI]: 1.32–1.90) than their counterparts living far from the borders. This result was robust to different choices of border delineation (i.e. ≤50, ≤75, ≤125, and ≤150 km). Women from poor families, those who have no access to improved toilets, reside in lowland areas, and are Muslim, were independently associated with underweight. In contrast, more wealth, higher education, older age, access to improved toilets, being married, and living in urban or lowlands were independently associated with overweight.

**Conclusions:**

The problem of undernutrition among women in Ethiopia is most worrisome in the border areas. Targeted interventions to improve nutritional status in these areas, such as improved access to sanitation, economic and livelihood support, are recommended.

## Introduction

The nutritional status of women has important implications both for their own health and that of their children. Malnutrition increases women's susceptibility to diseases and slow recovery from illnesses, and puts them at higher risk of pregnancy-related mortality. The children of undernourished women are more likely to be undernourished, which can lead to poorer cognitive development, shorter stature, and higher risk of morbidity and mortality. Malnutrition also hampers women's productivity and that of the society at large (1–3).

Women's undernutrition particularly remains a public health challenge in Ethiopia (4). Comparative studies conducted in Sub-Saharan Africa document the high prevalence of women's underweight in Ethiopia (5, 6). When compared to 57 low- to middle-income countries, Ethiopian women have the lowest average body mass index (BMI) (7). Various studies assessed the levels and identified the determinants of maternal malnutrition in Ethiopia using large-scale datasets, some of which focused on smaller geographical areas (8, 9), while others were representative of the entire country (6, 10).

Although health outcomes heavily depend on contextual factors such as community-based services and norms operating at community level (2), studies investigating women's nutritional status differentials in border areas are lacking. Border-related conflicts, common in the Horn of Africa (11–13), and neglected and poorly developed infrastructure and services at the borders (13, 14) have the potential to undermine the socioeconomic and health-related wellbeing of the populations (15). Moreover, the influx of refugees fleeing crisis in neighboring countries are mainly hosted in camps located in the border areas, which might impact the livelihood and health of the hosting communities (15). The assessment of health issues, such as nutritional status, is thus of particular relevance in countries like Ethiopia, which borders conflict zones (Somalia, South Sudan, Eritrea, and Sudan), and hosts a large population of refugees (16), while facing its own internal development challenges. This study therefore aims to assess the impact of living close to borders on the nutritional status of women in Ethiopia, while considering other important covariates.

## Methods

Our analysis was based on the third comprehensive Ethiopian Demographic and Health Surveys (EDHS) 2011 dataset. The survey was conducted to provide information mainly on fertility, family planning, HIV/AIDS, maternal and child health, and nutrition indicators at the national and regional levels. A stratified two-stage cluster sampling design was used to select the sample households. The sample initially included 624 enumeration areas (clusters) – 437 rural and 187 urban areas. However, in the Somali region, 28 of the 65 clusters were not interviewed due to drought and security concerns. Overall, 16,515 women of reproductive age (15–49 years) were selected from 596 clusters to form a representative sample of this age group. The data was collected using a questionnaire, which was adapted from the MEASURE DHS project (4).

**Fig. 1:**
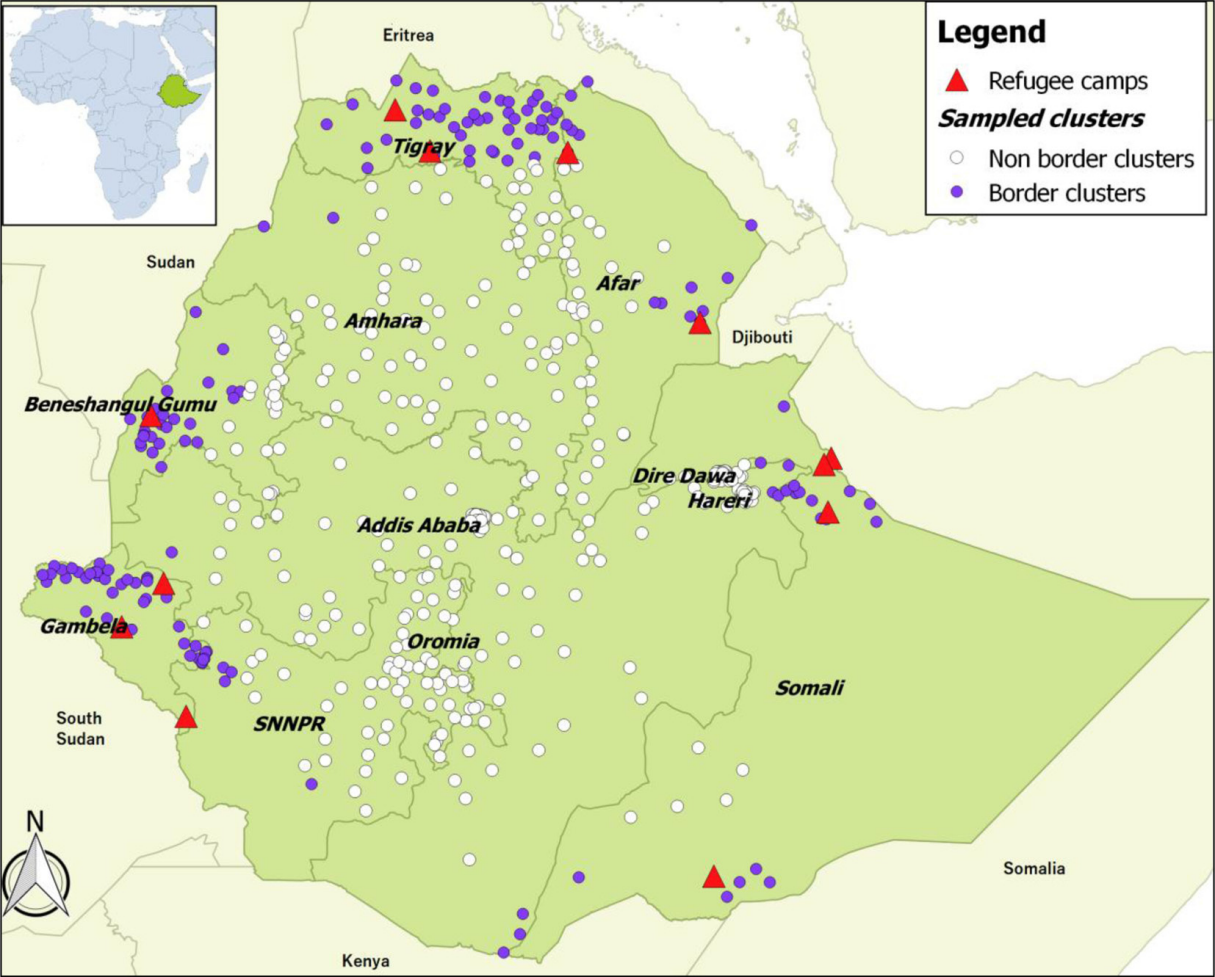
Map of Ethiopia showing EDHS sampled clusters and refugee camps together with neighboring countries.

BMI, defined as weight (in kilograms) divided by height (in meters) squared was used to assess the nutritional status of women. The use of BMI cut-offs is not recommended for adolescents (15–19 years) due to the remarkable growth during this period (1). Therefore, 3,835 adolescents were excluded from the analysis. Further, 5,714 pregnant and lactating women were excluded since BMI is not an appropriate indicator for this group either. We further removed 281 women due to missing weight or height, six women with flagged case records (implausible weight or height measurement), and 345 women without an attribution of geographical location. Finally, 6,334 adult women aged 20–49 years from 571 clusters were included in the analysis (Fig. 1).

### Outcome variable

The outcome variable is the women's nutritional status measured in BMI. We adopted the WHO BMI cut-offs (1), distinguishing between underweight (BMI<18.5 *kg*/*m* ^2^), normal weight (18.5–24.9 *kg*/*m* ^2^), and overweight (≥25.0 *kg*/*m* ^2^).

### Exposure variable

The exposure variable of the study is the residence status (border resident or not), measured by the nearest distance, in kilometers (km), from the borders to the sampled clusters. We used the GPS coordinates of the sampled clusters to measure the nearest distance to the border. Then, we dichotomized the distance into border (≤100 km) and non-border (>100 km), based on a previous study (17). We also assessed the sensitivity of our results to the different delineation of borders: ≤50, ≤75, ≤125, and ≤150 km.

### Confounding variables

The selection of confounding variables was guided by previous studies (5, 6, 8–10). These variables are woman's age (in full years); level of education (no education, primary, and secondary or above); marital status (never married, married or living together, and divorced or separated or widowed); religion (orthodox, protestant or catholic, and Muslim or others); household headship status (woman-headed or man-headed); household wealth (poor, middle, and rich); and place of residence (urban or rural). Toilet facility and source of drinking water were coded as improved or not improved based on the EDHS classification (4). Further, three agro-ecological zones were defined based on the altitude data: lowland (<1,500 meters), midland (1,500–2,300 meters), and highland (>2,300 meters) above sea level, as altitude has a strong influence on climate and thereby agriculture and livelihood.

### Statistical analysis

The EDHS data has a hierarchical structure, as women are grouped within clusters. This structure often yields data that are correlated and thus cannot be assumed independent. A multilevel approach adequately represents the unexplained variability of the nested structure, which is often difficult to represent in the single-level approach (18). Taking into account the hierarchical structure of the dataset and the possible correlation that may exist within and between clusters, we used a two-level random intercept multinomial logistic regression model.

In order to get unbiased estimates for the parameter in multinomial logistic regression, the Monte Carlo Markov Chain (MCMC) method was used as recommended by Browne (19). For the MCMC estimation, we used diffused prior distributions: normal priors with large variances (mean=0, variance=10^6^) for regression parameters and inverse gamma (0.001, 0.001) for precision parameters. We then run the chain for 50,000 iterations with a burn-in length of 25,000 iterations to ensure convergence of the Markov chains. The convergence of Markov chain was assessed by a visual inspection of the trace- and autocorrelation-plots. Further, Bayesian deviance information criterion (DIC) was used to evaluate the goodness-of-fit of the models (19).

Three models were fitted. Model 1 does not include any covariate; Model 2 include cluster level covariates (distance from the border, place of residence, and agro-ecological zones); and Model 3 include variables in Model 2 and individual level covariates. We also conducted a sensitivity analysis to assess whether the different choice of distance from the border might have impacted the results. We used survey weight as provided in the EDHS, to ensure the representativeness of the sample characteristics.

Finally, we reported the posterior odds ratio (OR) and 95% Bayesian credible intervals (CrI) for each of the variables in the full model (Model 3). The statistical significance was based on the non-inclusion of 1.0 in the 95% CrI. The statistical analysis was performed in STATA/IC 12.1 using *runmlwin* command, a program to run the MLwiN multilevel modeling software from within Stata (20). The map was produced using QGIS (21).

## Results

Table 1 presents the sample characteristics. The median women's BMI was 20.1 (interquartile range: 18.5–22.0 *kg*/*m* ^2^); nearly 25% of the women were underweight and 8% were overweight. The average distance between a border and a cluster was 184.4 km (ranging from 0.2 to 449 km). About 10% of the women lived close to the borders (i.e. ≤100 km) (Fig. 1). The median age was 33.0 (interquartile range: 25.0–41.0 years). Over half of the women (56%) were not educated; 20% were never married; 31% were from poor family backgrounds; 30% were household heads; and 52% were orthodox religion followers. The proportion of women who had access to improved toilet facilities and drinking water source were 22 and 59%, respectively. Most of the women (71%) were rural dwellers; and over one-tenth (12%) lived in lowland areas (Table 1).

**Table 1:** Sample characteristics of non-pregnant, non-lactating adult women aged 20–49 years in Ethiopia, EDHS 2011

Variables	*n*	% (95% CI)
BMI	6,334	20.1 (18.5, 22.0)^a^
Underweight		24.5 (22.9; 26.1)
Overweight		8.4 (7.4; 9.4)
Residence status	6,334	
Border residents		10.3 (9.5; 11.2)
Age (years)	6,334	33.0 (25.0, 41.0)^a^
Level of education	6,334	
No education		56.3 (54.4; 58.1)
Primary		28.4 (26.7; 30.1)
Secondary and above		15.3 (14.0; 16.7)
Marital status	6,334	
Never in union		19.8 (18.4; 21.3)
Married/living together		61.0 (59.1; 62.8)
Others^b^		19.2 (17.8; 20.7)
Religion	6,331	
Orthodox		51.9 (50.0; 53.7)
Protestant and catholic		22.7 (21.1; 24.3)
Muslim and others		25.4 (23.8; 27.1)
Household headship	6,334	
Women headed		30.4 (28.7; 32.2)
Wealth index	6,334	
Poor		31.4 (29.7; 33.1)
Middle		18.0 (16.6; 19.5)
Rich		50.6 (48.7; 52.5)
Drinking water source	6,152	
Improved		59.3 (57.4; 61.1)
Toilet facility	6,146	
Improved		21.6 (20.2; 23.1)
Place of residence	6,334	
Rural		71.4 (69.6; 73.1)
Agro-ecological zone	6,334	
Lowland		12.1 (11.1; 13.3)
Midland		54.4 (52.6; 56.2)
Highland		33.5 (31.8; 35.2)

CI (confidence intervals) are based on weighted sample.

a Median (interquartile range: 1st quartile (Q1), 3rd quartile (Q3)); Catholic and other religion followers were 1.1 and 0.6%, respectively

b widowed/divorced/separated.

Table 2 presents results from the random intercept Bayesian multilevel multinomial logistic regression model (Model 3). After controlling for potential confounders, the distance to a border was significantly associated with both underweight and overweight. The odds of being underweight were 59% higher among women living close to a border (OR=1.59; 95% CrI: 1.32–1.90), while the odds of overweight were 37% lower (OR=0.73; 95% CrI: 0.54–0.95) for similar comparisons. Table 3 shows the sensitivity analysis results for different choices of border definition. As we move closer to the borders, the odds of underweight increase (ranging from 35% higher odds for those who lived at 150-km distance from the border to 90% higher odds among those who lived within 50-km distance).

**Table 2:** Multilevel multinomial logistic regression model results for associations between individual- and cluster-level covariates with women's nutritional status, EDHS 2011

	Posterior OR (95% CrI)	
	
Variables	Underweight versus normal	Overweight versus normal
Residence status (versus non-border)		
Border residents	1.59 (1.32; 1.90)	0.73 (0.54; 0.95)
Age (years)	1.01 (1.00; 1.02)	1.07 (1.05; 1.08)
Level of education (versus secondary+)		
No education	1.05 (0.82; 1.33)	0.47 (0.35; 0.61)
Primary	0.92 (0.73; 1.14)	0.64 (0.51; 0.80)
Marital status (versus married)		
Never in union	1.15 (0.92; 1.41)	0.60 (0.45; 0.78)
Others	0.87 (0.72; 1.06)	0.82 (0.62; 1.07)
Religion (versus protestant)		
Orthodox	1.05 (0.85; 1.29)	0.97 (0.74; 1.26)
Muslim	1.35 (1.09; 1.65)	1.17 (0.86; 1.56)
Household headship (versus men headed)		
Women headed	1.08 (0.92; 1.27)	0.93 (0.74; 1.14)
Wealth index (versus rich)		
Poor	1.41 (1.15; 1.70)	0.46 (0.30; 0.67)
Middle	1.25 (1.00; 1.55)	0.36 (0.19; 0.59)
Drinking water source (versus improved)		
Not improved	1.06 (0.90; 1.24)	0.84 (0.59; 1.17)
Toilet facility (versus improved)		
Not improved	1.28 (1.05; 1.52)	0.61 (0.49; 0.75)
Residence (versus rural)		
Urban	0.90 (0.70; 1.14)	3.18 (2.28; 4.33)
Agro-ecological zone (versus midland)		
Lowland	1.32 (1.09; 1.60)	1.63 (1.27; 2.06)
Highland	0.96 (0.78; 1.17)	1.10 (0.87; 1.38)

**Table 3:** Sensitivity analysis: effect of different border definition on nutritional status, adjusted for the confounders

	Posterior OR (95% CrI)	
	
Variable	Underweight versus normal	Overweight versus normal
Residence status (versus non-border)		
Border (≤50 km)	1.90 (1.56; 2.33)	0.73 (0.51; 1.05)
Border (≤75 km)	1.67 (1.39; 2.00)	0.72 (0.53; 0.97)
**Border (≤100 km)**	**1.59 (1.32; 1.90)**	**0.73 (0.54; 0.95)**
Border (≤125 km)	1.36 (1.13; 1.56)	0.86 (0.67; 1.08)
Border (≤150 km)	1.35 (1.15; 1.60)	0.97 (0.77; 1.23)

In addition, the odds of underweight were significantly higher among women from poor families (OR=1.41; 95% CrI: 1.15–1.70) compared to those from rich families; among lowland women (OR=1.32; 95% CrI: 1.09–1.60) compared to midland women; among Muslim women (OR=1.35; CrI: 1.09–1.65) compared to protestant; and among women who had no access to improved toilet facilities (OR=1.28; 95% CrI: 1.05–1.52) (Table 2).

On the other hand, women who had no education (OR=0.47; 95% CrI: 0.35–0.61) or primary education (OR=0.64; 95% CrI: 0.51–0.80) were less likely to be overweight than women with secondary education or above. Similarly, women from poor families (OR=0.46; 95% CrI: 0.30–0.67) and middle class families (OR=0.36; 95% CrI: 0.19–0.59); who had no access to improved toilet facilities (OR=0.61; 95% CrI: 0.49–0.75); and who were never married (OR=0.60; 95% CrI: 0.45–0.78) were less likely to be overweight. The odds of being overweight were much higher among urban women (OR=3.18; 95% CrI: 2.28–4.33); and women who lived in lowland areas (OR=1.63; 95% CrI: 1.27–2.06). Finally, 1 year increase in age was associated to 7% higher odds of being overweight (95% CrI: 5–8%) (Table 2).

The variances (*Var*(*u*_1_) and *Var*(*u*_3_)) and covariance (*Cov*(*u*_13_)) estimates of the random effects parameters in Model 1 suggest that there is a large variation in women's nutritional status between clusters. The inclusion of all covariates (Model 3) reduced the between-cluster variances substantially. Moreover, the reduction of Bayesian DIC from Model 1 to Model 3 suggests that the full model best fit the data (Table 4).

**Table 4:** Cluster level random effects parameter estimates

Parameters	Model 1	Model 2	Model 3
*Var*(*u*_1_)	1.25 (0.89; 1.69)	0.18 (0.10; 0.29)	0.07 (0.01; 0.15)
*Var*(*u*_3_)	0.54 (0.40; 0.69)	0.31 (0.21; 0.43)	0.27 (0.16; 0.38)
*Cov*(*u*_13_)	−0.58 (−0.76; −0.42)	−0.22 (−0.30; −0.15)	−0.12 (−0.21; −0.06)
Bayesian DIC	10290.7	10104.4	9860.2

*Var*(*u*_1_) and *Var*(*u*_3_) are between-cluster variance between underweight versus normal and overweight versus normal, respectively; *Cov*(*u*_13_) cluster-level covariance.

## Discussion

Women's health, including an appropriate, sufficient, and balanced nutrition, is central to their development and empowerment and has positive implication for society as a whole. The fulfillment of women's nutritional needs is an issue of human development and not an isolated public health concern. However, our study reveals that about 25% of the (non-pregnant, non-lactating) adult women in Ethiopia were underweight while 8% were overweight. Such underweight prevalence is classified as serious according to the WHO Expert committee on physical status (1). Moreover, the odds of being underweight were 59% higher among women living in the border areas of the country.

Many factors can explain the border differentials. For example, the Afar, Gambella, Somali and Benishangul-Gumz regions in general and their peripheries in particular are poorly developed (12, 22). Recurrent drought, land degradation, a low level of livelihood diversification and coping strategies increased susceptibility to food crises in the Afar, Somali and Tigray regions (23, 24). Limited availability of health and other basic development infrastructures, coupled with conflicts and natural disasters occurring inside their borders (11–13), could contribute to the noticed high levels of underweight.

Furthermore, the border areas themselves have been struggling with their own internal and cross-border violent conflicts: for example, instability has affected the regions of Afar (25), Somali (26, 27), Gambella (28), Benishangul-Gumuz (27), and cross-regional clashes among Ethiopian pastoralists have also occurred (29). These often precipitate humanitarian crises, given the existing conditions of fragile livelihoods, limited community infrastructure and services (30), and may worsen women's health in the borders. Addressing internal and cross-border conflicts, through strengthening the indigenous systems and incorporating local perspectives for conflict resolution, and creating inclusive and strong government-community relations in conflict-prone borders, could help improve the livelihood of the community.

Ethiopia has also hosted a large number of refugees escaping conflict, political instability, inter-ethnic clashes, and droughts in neighboring countries (16). The majority of refugees are settled in camps located in the border zones: Somalia refugees who fled insecurity and famine following the collapse of the central government in 1991 are mainly located in the Somali region; Eritrean refugees seeking asylum after the Ethiopia-Eritrea war in 1998 are hosted in the Tigray and Afar regions; South Sudanese refugees who have fled inter-ethnic clashes, since 1991, are hosted in the Gambella region; and Sudanese refugees fleeing from the Blue Nile and Darfur conflicts are settled in the Benishangul-Gumz region (Fig. 1). Some refugees also live with host communities. Refugees bring both benefits and challenges for the communities that receive them (31, 32). Disease outbreaks, food scarcity, overburden of health facilities, security problems, and environmental degradation are among the burdens refugees could bring to the already fragile hosting community (15, 32). For instance, a study conducted in Tanzania documented price increases in non-aid food items in refugee hosting areas (33), while another study reported the increased incidence of malaria in refugee-receiving countries (34). Thus, hosting refugees cannot be discarded as an additional contributor to the observed high prevalence of underweight among women residing close to the borders. At the same time, the presence of refugee camps can contribute to market development and increase access to the health services provided in the camps (31, 32). Therefore, integrating host communities in humanitarian interventions for refugees is crucial to increase acceptance and strengthen the local response capacity. Further research is needed to understand the pathways through which women's health is affected by the presence of refugees.

Our results further depicted that lowland women were more likely to be underweight. In the lowland areas of Ethiopia, the communities are mainly pastoralist and semi-pastoralist (24). Population growth, increased competition for scarce resources, loss of crop and livestock productivity due to natural disasters, and poor infrastructure development heightens the vulnerability of pastorals livelihood (24, 25), which may have exacerbated the nutrition problems in the area. In addition, the low dietary diversity and calorie-poor pastoralist diet (35), and vector-borne diseases like malaria and sleeping sickness in lowlands may directly or indirectly contribute to women's underweight (35, 36). Pastoral communities would benefit from targeted interventions to improve access to water and health care for themselves and their livestock, implement diet diversification programs, and strengthen access to market and livestock trade.

It is well recognized that poverty affects an individual's health status by various channels, be it through household food security, health care utilization, access to sanitation facilities, or gender inequality (37). Our study shows that women from poor families are more likely to be underweight, but less likely to be overweight. Our results are in line with similar studies conducted in Ethiopia and other developing countries (6, 9, 38–40). Lack of improved toilet facilities is a proxy indicator for poor hygiene (41), and poor hygiene in turn increases the risk of diseases that can aggravate undernutrition. As expected, our results portrayed that women with no access to improved toilet facilities had higher chances of being underweight. Muslim women were more likely to be underweight: one possible reason might be the high fertility rate and shorter birth-interval of Muslim women (42), which worsens maternal nutritional status (43).

We found that the odds of overweight increased with age, education, family wealth, having access to improved toilet facilities, and living in urban settings or lowlands. Studies conducted in Ethiopia and other developing countries corroborate our findings (9, 39, 40, 44), where the above factors tend to be associated with overweight and obesity through reduced physical activity, increased sedentary lifestyle, and increased consumption of high calorie foods and animal products. Further exploration of our data revealed that access to improved toilet facilities defines an urban profile, where overweight is more likely to occur. On the other hand, due to recurrent droughts, the Ethiopian lowlands are the primary recipients of large-scale humanitarian aid (29), and relying on food assistance as the main source of food is a risk factor for overweight (45). Further, Dire Dawa and part of the Harari regions are located in the lowlands, which are predominantly urban areas where overweight tends to be higher. Finally, the lower odds of overweight among never-married women could be because these women are more likely to be younger and less educated, both of which are protective factors against overweight. In sum, the problem of overweight should not be overlooked, given the well-acknowledged double burden of under- and over-nutrition currently facing developing countries (44).

Our study presents some limitations. First, BMI is influenced by the body shapes found in a population with heterogeneous body proportions (the ratio of leg-length to torso-length) (1). As the EDHS dataset lacks a sitting length that can be used to standardize the BMI, we used the BMI provided in the survey. Second, since cluster-level characteristics, such as community-level poverty, education, and access to toilet facilities, were not collected in EDHS, we included only three cluster-level covariates in our analysis (place of residence, agro-ecological zones, and distance to the border). Third, the cross-sectional nature of the data allows inferences on associations, but precludes establishing causal links. Finally, but important, our study only addressed one health component, the nutritional status in women 20–49 years of age. The prevalence of other diseases is also relevant, and other vulnerable groups such as children could also be affected by the border differentials.

## Conclusions

Women's malnutrition, particularly undernutrition, remains an important public health challenge in Ethiopia, which is not tackled adequately to date. The burden is particularly heavy among women who live close to the borders. Targeted interventions that are recommended to improve nutritional status in borders zones include improved access to sanitation, and programs to enhance household wealth and livelihood support. Our results also acknowledge pockets of overweight particularly in urban and affluent families, which should not be overlooked. Finally, our study aims to foster the debate on the health of communities living at the borders by investigating both internal and external factors. In view of the growing trends in internal and cross-border insecurity both in Ethiopia and elsewhere in the region, this type of study provides important insights into identifying vulnerable population groups and zones.

## Paper context

Ethiopian borders are poverty-stricken and are regularly rampaged by conflict and refugee flows, which might ultimately impact health. However, studies investigating the influence of living close to an international border on women's nutrition are lacking. Our study indicates that the burden of women's undernutrition is heavy in the border areas of Ethiopia. We recommend interventions, such as improved access to sanitation, and economic and livelihood supports to improve nutritional status in these areas.

## References

[1] WHO (1995). Physical status: the use and interpretation of anthropometry.

[2] WHO (2005). The World Health Report 2005: make every mother and child count.

[3] Black RE, Victora CG, Walker SP, Bhutta ZA, Christian P, De Onis M (2013). Maternal and child undernutrition and overweight in low-income and middle-income countries. Lancet.

[4] Central Statistical Agency [Ethiopia] and ICF International (2012). Ethiopia Demographic and Health Survey 2011.

[5] Mukuria A, Aboulafia C, Themme A (2005). The context of women's health: results from the demographic and health surveys, 1994–2001. Comparative Reports.

[6] Bitew FH, Telake DS (2010). Undernutrition among women in Ethiopia. DHS working papers.

[7] Corsi DJ, Finlay JE, Subramanian SV (2012). Weight of communities: a multilevel analysis of body mass index in 32,814 neighborhoods in 57 low- to middle-income countries (LMICs). Soc Sci Med.

[8] Regassa N, Stoecker BJ (2012). Contextual risk factors for maternal malnutrition in a food-insecure zone in Southern Ethiopia. J Biosoc Sci.

[9] Tebekaw Y, Teller C, Colón-Ramos U (2014). The burden of underweight and overweight among women in Addis Ababa, Ethiopia. BMC Public Health.

[10] Girma W, Genebo T (2002). Determinants of nutritional status of women and children in Ethiopia.

[11] Weber A (2012). Boundaries with issues: soft border management as a solution? Friedrich-Ebert-Stiftung, Africa Department, Berlin, Germany. http://library.fes.de/pdf-files/iez/08869.pdf.

[12] Feyissa D, Hoehne MV (2008). Resourcing state borders and borderlands in the horn of Africa. Report No: 107.

[13] Markakis J Borders and borderland communities in the horn: the failure of integration.

[14] Institute of Security Studies (2012). Africa's international borders as potential sources of conflict and future threats to peace and security.

[15] Baez JE (2011). Civil wars beyond their borders: the human capital and health consequences of hosting refugees. J Dev Econ.

[16] UNHCR (2014). UNHCR Global Report 2014: hosting the world's refugees.

[17] Cogneau D, Mesple-somps S, Spielvogel G (2010). Development at the border: a study of national integration in post-colonial West Africa. G-MonD Working Paper.

[18] Goldstein H (2011). Multilevel statistical models.

[19] Browne WJ (2014). MCMC estimation in MlwiN, version 2.31. Center for multilevel modeling.

[20] Leckie G, Charlton C (2012). runmlwin: a program to run the MlwiN multilevel modeling software from within Stata. J Stat Softw.

[21] QGIS Development Team (2015). QGIS Geographic Information System. Open Source Foundation Project.

[22] Central Statistical Agency (2012). Statistical abstract. http://www.csa.gov.et/.

[23] Lanz TJ (1996). Environmental degradation and social conflict in the Northern Highlands of Ethiopia: the case of Tigray and Wollo Provinces. Afr Today.

[24] Pantuliano S, Wekesa M (2008). Improving drought response in pastoral regions of Ethiopia: Somali and Afar regions and Borena Zone in Oromiya region.

[25] 
Rettberg S (2010). Contested narratives of pastoral vulnerability and risk in Ethiopia's Afar region. Pastoralism.

[26] Devereux S (2006). Vulnerable livelihoods in Somali Region, Ethiopia. IDS Research Report.

[27] Adegehe AK (2009). Federalism and ethnic conflict in Ethiopia: a comparative study of the Somali and Benishangul-Gumuz regions (Doctoral Thesis). http://hdl.handle.net/1887/13839.

[28] Dereje F, Ege S, Aspen H, Teferra B, Bekele S (2009). A national perspective on the conflict in Gambella. Proceedings of the 16th International Conference of Ethiopian Studies.

[29] Hagmann T, Mulugeta A (2008). Pastoral conflicts and state-building in the Ethiopian Lowlands. Africa Spectr.

[30] World Bank (2011). World development report 2011: conflict, security, and development.

[31] Meyer S, Tappis H, Weiss W, Spiegel P, Vu A (2011). Refugee site health service utilization: more needs to be done. Am J Disaster Med.

[32] Jacobsen K (2002). Can refugees benefit the state? Refugee resources and African state building. J Mod Afr Stud.

[33] Alix-Garcia J, Saah D (2009). The effect of refugee inflows on host communities: evidence from Tanzania. World Bank Econ Rev.

[34] Montalvo JG, Reynal-Querol M (2007). Fighting against malaria: prevent wars while waiting for the ‘miraculous’ vaccine. Rev Econ Stat.

[35] Sadler K, Kerven C, Calo M, Manske M, Catley A (2009). Milk matters: a literature review of pastoralist nutrition and programming responses.

[36] Demissie T, Mekonen Y, Haider J (2003). Agroecological comparison of levels and correlates of nutritional status of women. Ethiop J Heal Dev.

[37] WHO (2003). Poverty and health.

[38] Neuman M, Finlay JE, Smith GD, Subramanian SV (2011). The poor stay thinner: stable socioeconomic gradients in BMI among women in lower- and middle-income countries. Am J Clin Nutr.

[39] Subramanian SV, Smith GD (2006). Patterns, distribution, and determinants of under- and overnutrition: a population-based study of women in India. Am J Clin Nutr.

[40] Kandala NB, Stranges S (2014). Geographic variation of overweight and obesity among women in Nigeria: a case for nutritional transition in Sub-Saharan Africa. PLoS One.

[41] Mara D, Lane J, Scott B, Trouba D (2010). Sanitation and health. PLoS Med.

[42] Teller C, Gebreselassie T Religious, ethnic, and regional factors of high fertility in Ethiopia. Popul Ref Bur 2009.

[43] Conde-Agudelo A, Rosas-Bermúdez A, Castano F, Norton MH (2012). Effects of birth spacing on maternal, perinatal, infant, and child health: a systematic review of causal mechanisms. Stud Fam Plann.

[44] Popkin BM, Adair LS, Ng SW (2012). Now and then: the global nutrition transition: the pandemic of obesity in developing countries. Nutr Rev.

[45] Grijalva-Eternod CS, Wells JCK, Cortina-Borja M, Salse-Ubach N, Tondeur MC, Dolan C (2012). The double burden of obesity and malnutrition in a protracted emergency setting: a cross-sectional study of western Sahara refugees. PLoS Med.

